# A cross-sectional investigation of psychosocial stress factors in German families with children aged 0–3 years during the COVID-19 pandemic: initial results of the CoronabaBY study

**DOI:** 10.1186/s13034-022-00464-z

**Published:** 2022-05-17

**Authors:** Catherine Buechel, Ina Nehring, Clara Seifert, Stefan Eber, Uta Behrends, Volker Mall, Anna Friedmann

**Affiliations:** 1 Technical University of Munich, TUM School of Medicine, Chair of Social Pediatrics, Munich, Germany; 2 Professional Association of Pediatricians in Bavaria and PaedNetz Bayern, Munich, Germany; 3 Department of Pediatrics, Kinderklinik München Schwabing, StKM GmbH and Klinikum rechts der Isar, Technical University of Munich, Munich, Germany; 4 Heiglhofstraße 65, 81377 München, Germany

**Keywords:** Parent psychosocial functioning, Behavior problems, COVID-19, Depression, Anxiety, Early life adversity, Infancy and early childhood, Parenting, Psychosocial functioning, Stress

## Abstract

**Background:**

Psychosocial stress during the COVID-19 pandemic is increasing particularly in parents. Although being specifically vulnerable to negative environmental exposures, research on psychosocial stress factors in infants’ and toddlers’ families during the pandemic is so far sparse. The CoronabaBY study investigates the perceived pandemic burden, parenting stress and parent and child mental health problems in families with children aged 0–3 years in Bavaria, Southern Germany. Further, the relationships between these psychosocial stressors are examined and sociodemographic characteristics that may be predictive of these factors will be explored.

**Methods:**

Participants were cross-sectionally surveyed via smartphone app. Standardized questionnaires on perceived pandemic burden, parenting stress, parental symptoms of depression and anxiety, infants’ crying, sleeping and feeding problems or toddlers’ emotional and behavioral problems were applied.

**Results:**

*N* = 991 parents (*M*_age_ = 33.7 years, *SD* = 4.5; 93.7% mothers, 91.5% born in Germany) with infants (*n* = 554; *M*_age_ = 5.9 months, *SD* = 3.0) or toddlers (*n* = 435; *M*_age_ = 25.9 months, *SD* = 6.5) participated in the first half-year of 2021. Sixty-five percent of the parents perceived a high pandemic burden, 37.7% experienced parenting stress and 24.1% showed affective symptoms (anxiety: 30.1%, depression: 18.5%). Feeding problems, crying/ sleeping problems and multiple regulatory problems were found in 34.8%, 26.2% and 13.5% of the infants, respectively. Amongst toddlers, 8.5% showed noticeable behavior and emotional problems. Children`s mental health problems correlated moderately with parenting stress and parental affective symptoms and weakly with perceived pandemic burden. A lower financial status, higher parental education and increasing child age were significant but weak predictors for higher parenting stress, affective symptoms and higher psychological problems in children.

**Conclusions:**

A majority of the surveyed families with infants and toddlers experience the pandemic as stressful. The main challenges are parental affective symptoms and limited resources for childcare due to parenting stress. Overall, infants and toddlers show similar levels of mental health problems when being compared to pre-pandemic studies, but staggered detrimental effects on children`s mental health might occur if the stressful conditions persist. This is already indicated by correlations between parental and child psychosocial stress factors.

## Introduction

Longitudinal studies over recent decades clearly demonstrate the potentially harmful influence of early psychosocial stress on children's mental health, which can have an impact across the entire lifespan [1, 2]. Psychosocial stress can derive from a family’s challenging living conditions (e.g., low socioeconomic status, lack of support, social isolation), strained relationships (e.g., parent-child relationship) and demanding family characteristics (e.g., family health problems, limited resources for childcare) [3].

In the face of the COVID-19 pandemic, psychosocial stress is increasing and has been found to be particularly pronounced within families [4–6]. Alongside the fear of infection, COVID-19 restriction measures with their potential of causing social isolation [7], negative economic changes [8], additional childcare responsibilities [5], disruptions in everyday routines and limited access to family support services [9] have a significant impact on family and child wellbeing.

So far, the majority of recent studies focus on the impact of COVID restriction measures on school children and adolescents, who show significantly more psychological problems than children in pre-pandemic studies [10]. However, very young children’s mental health and their family’s wellbeing have so far been more or less neglected in research during the pandemic. This is to some extent startling, as families with very young children are generally known to be specifically vulnerable: the first years of a child's life are exceptionally demanding because of their exclusive dependency on the parents’ physical and emotional care and protection, which requires continuous supervision and extensive parental involvement. Accordingly, having younger children is related to higher parenting-related exhaustion [11]. At the same time, the resilience of infants and toddlers in the face of negative environmental influences (e.g., elevated parenting stress, parental psychopathology) is not yet strongly developed [12]. Overall, 0–3-year-old children are considered a specific risk group for the potential negative effects of psychosocial stress, which in extreme cases (e.g., parental burnout) can result in neglect or maltreatment [13]. Therefore, knowledge about psychosocial stressors in this vulnerable group during particularly challenging times such as the current pandemic is crucial for implementing appropriate interventions to maintain infants' and toddlers' mental health and to prevent potential long-term consequences for their development. As very young children are exceptionally dependent on the relationship with their caregivers, their well-being is closely intertwined with that of their parents. This link may have even intensified as COVID-19 pandemic measures — including limited access to nurseries and the potential absence of alternative caregivers within the family (such as grandparents) due to physical distance measures — have led to many children spending more time at home with their parents.

Hence, infants and young children might be less influenced by the structural and societal changes imposed by the pandemic, but rather indirectly affected by the related psychosocial stresses on their parents, which could impact parenting resources. The so far scarcely available findings on psychosocial stress in families with infants and toddlers already indicate that the existential worries triggered by the COVID-19 pandemic are particularly pronounced in this group [5]. Parents of very young children experienced the largest decreases with regard to general life and family satisfaction during the pandemic when being compared to families with older children [5]. Generally, parents are reported to experience the pandemic as more stressful than individuals without children [5, 14].

For parents, a high ‘pandemic burden’ (their perception of living conditions under COVID as very stressful) risks limiting their ability to fulfill the caregiving role, especially if additional challenges, e.g., increased childcare demands or parental mental health problems are present. In fact, several studies report a substantial increase in parenting stress — an adverse psychological reaction to parents’ resources and childcare demands being out of balance [15] — related to the pandemic [16–18]. Literature has repeatedly emphasized the crucial role of parenting stress as a risk factor for child mental health problems. Parenting stress is linked to negative parent-child interactions [19, 20] — especially with regard to limited parental emotional availability and the ability to sensitively react to a child’s needs [19–21] —, to a lower quality of caregiving in general, e.g., more harsh parenting styles [21], and a higher risk of child maltreatment and neglect [22]. Hence, parenting stress as a psychosocial risk factor has a significant importance for child mental health in general [23, 24] and also especially during the current pandemic [25].

Parental mental health problems are another psychosocial risk factor for the development of mental health problems in children [26–28]. Parental mental health problems, more specifically depression and anxiety, are additionally closely linked to parenting stress [19, 29, 30]. During the COVID-19 pandemic, heightened depression and anxiety rates have been found in parents [6], especially for mothers [31], with negative implications for parent-child relationships [32, 33].

As mentioned earlier, families with children aged 0–3 years are generally considered to be particularly vulnerable to adverse environmental exposures as present during the COVID-19-pandemic. However, to be able to provide adequate interventions to families in actual need of support, specific risk groups defined by certain sociodemographic characteristics within this population are still to be identified. Studies during the pandemic have found low parental education to be associated with stronger mental health problems in older children and adolescents [34] and low household income to be associated with higher burden in parents of school-age children [14]. Pre-pandemic data on families with infants and toddlers show an impact of the child`s age and the number of children for parental wellbeing. Accordingly, many psychosocial stress factors for parents increase in the course of early childhood [35]. Additionally, having more children is related to more parenting stress [36].

Against this background, we cross-sectionally explored the presence and extent of parent and child psychosocial stress factors in German families with children aged 0–3 years during a phase of the COVID-19 pandemic when many restriction measures were in effect. More specifically, the study pursues three objectives:

- 1. To examine the current rates of the perceived pandemic burden, parenting stress, parental anxiety and depression symptoms and infants’ crying, sleeping and feeding problems or toddlers’ emotional and behavioral problems that are evident during the pandemic.
- 2. To investigate the relationship between perceived pandemic burden and parent and child psychosocial factors as well as between the parental and child psychosocial factors.
- 3. To identify sociodemographic characteristics that might be predictive for the investigated psychosocial stress factors.

## Methods

### Study design

The ‘CoronabaBY’ study exploratively investigates intermediate and long-term psychosocial stress during different phases of the pandemic (‘Corona’) in families with infants and toddlers (‘baby’) in Bavaria (‘BY’). In this paper, we present results of the cross-sectional observation. Data were collected between February and June 2021 (second and third COVID-19 infection wave in Bavaria), when many COVID-19 restriction measures were still overall in effect. The study protocol was approved by the Ethics committee of the Technical University of Munich (vote no. 322/20 S) and pre-registered in OSF (https://osf.io/search/?q=tksh5&page=1).

### Participants

All participants were recruited and surveyed via smartphone app ‘Mein Kinder- und Jugendarzt’ (‘My pediatrician’) (www.monks-aerzte-im-netz.de). The app is a well-established communication tool that connects parents with their pediatrician. In a two-step recruitment procedure, all pediatricians in Bavaria (Southern Germany) using ‘My pediatrician’ as part of their practice management were invited to participate in the study (*N* = 300). After giving informed consent (*N* = 73, response rate = 24.3%), an invitation for study participation was sent out via app to all eligible patients of the participating pediatricians. All parents of children between 3 months and 3 years who used the app and who understood the German study invitation were eligible to take part. The study invitation and detailed information as well as the informed consent form were presented via app. 5317 invitations were sent out via push-message. According to digital user behavior analysis, about 55% of the app using parents read this message. Of these, 1115 (approx. 37%) gave informed consent and 991 (approx. 33%) completed the questionnaires. To minimize potential selection bias, the following measures were taken: in order to enhance acceptance of ‘CoronabaBY’ among pediatricians, we collaborated with the professional association of pediatricians, who supported the promotion of the study and personally contacted all eligible pediatricians. To bring additional attention to the study, ‘CoronabaBY’ was advertised via several channels (e.g., video tutorials posted on the professional association’s website, presentations held by the study team during pediatric roundtables). In order to reach as many families as possible, we kept the study invitation and participation for the families as simple as possible. Families received incentives for their participation.

### Measures

All data were collected via app using standardized questionnaires. Participants were asked questions on general sociodemographic characteristics, such as child’s gender and birthday, age of participant, country of origin, relation to the child (mother/father), highest degree of education, number of children living in the family and perceived financial status. We further posed questions on perceived pandemic burden and on additional psychosocial stress factors of parents (parenting stress, symptoms of depression and anxiety) and children (infants’ crying, sleeping and feeding problems or toddlers’ emotional and behavioral problems).

#### Pandemic-related restrictions and perceived pandemic burden

Overall, ten questions were asked about specific restrictions and perceived burden related to the pandemic (e.g., restrictions of social contacts, leisure activities and family support services, increased conflicts within the family). The perceived ‘pandemic burden’ for parents and children was derived from the 5-point-answer (from 1 = *not at all stressful* to 5 = *very stressful*) to the global question: ‘Taken together, what do you think: how stressful is/was the COVID-19 pandemic for you (please think of measures like social restrictions but also your personal experiences, related worries etc.)?’ and ‘Taken together, what do you think: how stressful is/was the COVID-19 pandemic for your child?’, respectively. Weak to moderate significant correlations (range: ρ = 0.138 to 0.378, *p* < 0.001) between the specific restrictions and the overall perceived burden were found.

#### Parenting stress

Parenting stress was assessed with the German Version of the ‘Parenting Stress Index (PSI)’ *(“Eltern-Belastungs-Inventar” EBI*; [37]), which is based on the parenting stress model by Abidin [15, 38] and includes a child domain (stress due to characteristics and behavior of the child) and a parent domain (detecting potential impairment of parental functions). High scores indicate limited parental resources for upbringing and care for the child. We applied the seven subscales of the parent domain: ‘health’ (health impairment as a cause or a result of parenting stress), ‘isolation’ (lacking integration in social networks), ‘role restriction’ (perceived limitations as a result of being parent), ‘parental competence’ (parental doubt about their own abilities to manage upbringing and care for their child), ‘attachment’ (emotional relation of parent on the child), ‘depression’ (limited emotional availability within the parent-child-relationship) and ‘spouse related stress’ (as a result of being a parent). Answers are given on a 5-point Likert scale ranging from 1 = *strongly agree* to 5 = *strongly disagree* resulting in a possible score range of 28 to 140. Three cut-off categories for each subscale and the whole parent domain can be applied: *no findings* (T-value < 60), *stressed* (T-value = 60–69), and *strongly stressed* (T-value ≥ 70) [37]. Internal consistency of the parent domain is good (α = 0.93). Retest reliability after one year is 0.87. The test of validity by means of correlations with stress indicators and related constructs resulted in the assumption of test validity [39].

#### Parental depression and anxiety symptoms

Symptoms of parental state (‘How do you feel right now?’) depression and anxiety were assessed with the State-Trait-Anxiety-Depression Inventory (STADI; [40]). The questionnaire includes the four subscales ‘emotionality’, ‘worry’, ‘anhedonia’ and ‘dysthymia’. Answers are given on a 4-point scale ranging from 1 = *not at all* to 4 = *very much*, resulting in a possible score range of 20 to 80. Based on age- and sex-dependent standardized cut-off T-values, each domain (‘depression’, ‘anxiety’, ‘total’) can be defined by symptoms to be *far below average* (T- value < 30)*, below average* (T-value = 30–39)*, average* (T-value = 40–60)*, above average* (T-value = 61–70), *far above average* (T-value > 70). Internal consistency of the global State-Scales (α = 0.92), the State-Depression-Scale (α = 0.87) and the State-Anxiety-Scale (α = 0.90) is good. Validity can be assumed based on comparison with other test procedures [41].

#### Infants’ crying, sleeping and feeding problems and toddlers’ emotional and behavioral problems

We applied the Questionnaire for Crying, Sleeping and Feeding (CSF; [42, 43]), which consists of 3 subscales (‘crying, whining, sleeping’, ‘feeding’ and ‘coregulation’). The coregulation subscale was not used in the present study. To assess infants’ (age 0–16 months) crying, sleeping and feeding problems, parents were asked 38 questions on behaviors in their infants. Answers were given on 4-point-scales and mean values were calculated (ranging from 1 to 4). According to validated cut-off values, the dichotomous outcome *noticeable problems* and *no problems* can be calculated for the domains: ‘crying, whining, sleeping’ (cut-off value: 1.84, sensitivity: 87%, specificity: 92%) and ‘feeding’ (cut-off value: 1.27, sensitivity: 57%, specificity: 77%). The CSF also comprises questions to identify excessive crying as defined by the Wessel criterion (‘rule of threes’) [44]. Furthermore, the CSF asks how stressful parents experience their children’s crying, sleeping and feeding behavior. The validity of the questionnaire is considered to be secured by the proof of high internal consistencies of the scales as well as by correlations with behavior diaries and clinical diagnoses [42].

For toddlers (age 17 months or older), we used the Strengths and Difficulties Questionnaire (SDQ, short form of the German Version; [45]) to examine emotional and behavioral problems. Parents are asked to classify the individual characteristics to be *not true*, *somewhat true* or *certainly true* for their child in four domains (‘emotional symptoms’, ‘conduct problems’, ‘hyperactivity/inattention’, and ‘peer relationship problems’). A score range of 0 to 40 points can be received. Corresponding to cut-off values, child behavior can be categorized as *normal* (0–13 points), *borderline* (14–16 points) or *abnormal* (17–40 points*)*. Internal consistency ranges between α = 0.73 and α = 0.86. By means of comparison with other corresponding scales (e.g., Child Behavior Checklist), the validity of the instrument can be assumed [46, 47].

### Statistical analyses

Since submission of questionnaires was only possible when all items were completed, we had only a few missing values because of obvious misreporting of parental age (e.g., 1 year). Subjects with missing values were left out for our analyses where necessary (see individual table descriptions).

Spearman’s Rho was calculated to identify potential correlations between the variables pandemic burden, parenting stress (EBI total score), parental depression and anxiety symptoms (STADI State total score), infants’ crying, whining and sleeping problems or feeding problems (according to corresponding subscales of CSF) or toddlers’ emotional and behavioral problems (SDQ total score), respectively.

We dichotomized education status into *high* (university degree and high school diploma) and *low* (secondary and lower secondary school diploma) and excluded those cases from our analyses whose response referred to ‘other qualification’ thus could not be allocated to either of the groups.

Financial status, as measured by participants' subjective perception that additional purchases are possible after basic needs are met, was also dichotomized into high (‘family income generally allows very large additional purchases’ and ‘family income generally allows for large additional purchases’) and low (‘family income generally only allows for small additional purchases’, ‘family income generally only allows for very small additional purchases’, ‘family income not sufficient to meet basic needs (no additional purchases possible)’. Participants who did not want to give an answer regarding their current financial status were excluded for the specific analyses related to this aspect.

For a multiple logistic regression model, we dichotomized pandemic burden into *high* (points 4 and 5 of Likert scale) or *low* (points 1 to 3 of Likert scale) as outcome variable.

Stepwise multiple linear regression models with education status, financial status, child age, child gender and having more than one child (respectively having a sibling) as predictor variables and EBI total score, STADI total score, CSF crying/sleeping problem score, CSF feeding problem score and SDQ total score as outcome variables were conducted. The model resulted in the calculation of standardized beta weights and their p-value for corresponding predictor variables. Requirements for calculating multiple linear regression models were complied. As there are specific STADI norms for women and men, we ran two linear regression models for this outcome. We put the same predictor variables into a logistic regression model with ‘parental pandemic burden’ as outcome resulting in odds ratios (OR) and corresponding 95% confidence (95% CI) intervals. All described results were based on an alpha level of 5%. Analyses were performed in IBM SPSS Statistics Version 28.0 for Windows.

## Results

### Sample characteristics

The final sample included 991 study participants. Mean parental age was 33.7 years (*SD* = 4.5), for mothers it was 33.3 years (*SD* = 5.0) and for fathers 37.4 years (*SD* = 5.1). Ninety-four percent of the participants were mothers. Half of the children (55%, n = 549) were < 12 months old, 24% (n = 241) were 13–23 months old, and 20% (n = 201) were ≥ 24 months of age. Mean infant age was 5.9 months (*SD* = 3.0, range: 1 to 13 months) and toddlers were averagely 25.9 months old (*SD* = 6.52, range: 17 to 37 months). Fifty-five percent of the children had siblings. More than half of the parents had at least high school diploma (Table 1). A perceived high financial status (before the pandemic) was reported by 58% of the parents. Sixty-five percent of the participants were on maternity/parental leave. Most of the parents (92%) were of German origin (Table 1).

**Table 1:** Sample characteristics

Parents	*%*	*n*
Mothers	93.7	929
Born in Germany	91.5	907
Mother tongue German	92.6	918
Maternity/parental leave	64.5	639
Level of education		
University degree	40.7	403
High school diploma	18.3	181
Secondary school diploma	30.8	305
Lower secondary school diploma	8.1	80
Other qualifications	2.2	22
Financial status (before pandemic)		
Very large additional purchases possible	11.3	112
Large additional purchases possible	46.2	458
Small additional purchases possible	28.9	286
Very small additional purchases possible	6.0	59
No additional purchases possible	1.1	11
Not specified	6.6	65

Children	*%*	*n*
1st year of life	55.4	549
2nd year of life	24.3	241
3rd year of life	20.3	201

Children	*%*	*n*
Boys	51.6	511
Chronic illness and/or disability	7.7	76

*N* = 991

### Presence and extent of psychosocial stress factors

#### Pandemic-related restrictions and perceived pandemic burden

The pandemic was perceived to be overall ‘stressful’ (‘very stressful’) for 43.2% (21.8%) of the parents. This was also true for 21.2% (15.5%) of the children (parent report). Individual restrictions and changes in living conditions related to the pandemic are shown in Tables 2 and 3.

**Table 2:** Perceived COVID-19-related restrictions

Kind of restriction	%	%	%	%	%
	1 (No perceived restrictions)	2	3	4	5 (High level of perceived restrictions)
Parent social contacts	0.9	3.5	20	47.6	28
Child social contacts	4	11.2	20.6	35.1	29.1
Family support services	2.6	5.1	17.3	31.9	43.1
Leisure activities	0.6	0.6	5.2	24	69.5

	1 (none)	2	3	4	5 (high)
Changes in childcare situation	33.8	10.9	13.2	18.2	23.9
Increased family conflicts	29.9	29.6	24.3	11.8	4.4
Worries about COVID- infections	9	15.3	28.5	27.2	20

Overall perceived pandemic burden	1 (not at all stressful)	2	3	4	5 (very stressful)
Parent	0.5	6.3	28.3	43.2	21.8
Child (parent report)	14.9	21.8	26.5	21.2	15.5

**Table 3:** Changes in occupational or financial situation during pandemic

Responding options	No changes	Partly/completely home office	Reduction of work hours	Job loss
Types of changes	%	%	%	%
Regarding own occupational situation^a^	35.1	45.8	12.3	0.8
Regarding spouse occupational situation^a^	47	35.6	10.4	1.2

Responding options	High financial burden due to pandemic	Medium financial burden due to pandemic	Little financial burden due to pandemic	No financial burden due to pandemic
Types of changes	%	%	%	%
Regarding the financial situation^b^	1.5	6.5	17.5	72.4

*N* = 991

^a^participants who selected the option “other” are not shown

^b^participants who did not want to answer are not shown

#### Parenting stress

The EBI showed 29.8% of the parents to be ‘stressed’ and 7.9% to be ‘strongly stressed’ (see Table 4). Looking at the individual subscales of the EBI in the overall sample, ‘depression (limited parental emotional availability)’ and ‘health’ stood out with 60.2% and 41.5% of participants scoring above the cut-off value for being ‘stressed’ or ‘strongly stressed’, respectively. According to the CSF (parents of infants only), 19.5% felt fairly ‘stressed’ to ‘strongly stressed’ by their child's crying and whining, 15.2% by their child's sleeping behavior and 3.1% by their child's eating behavior (see Table 4).

**Table 4:** *Parenting Stress according to EBI (N* = *991) and CSF (N* = *554)*

Parenting Stress Inventory (EBI)	%	n
* Categorial evaluation of the parent domain*^*a*^		
No findings	62.2	605
Stressed	29.8	290
Strongly stressed	7.9	77
* Subscales (above cut-off)*		
Attachment	23.8	236
Isolation	41.4	413
Parental competence	31.2	309
Depression (limited emotional availability in the parent-child-relationship)	60.2	597
Health	41.5	411
Role restriction	35.4	351
Spouse related stress^a^	40.6	395

Parenting stress due to crying, feeding and sleeping problems of the child (CSF)^b^	%	n
* Stressed due to crying and whining of the child*		
A little to not at all	80.5	446
Fairly to very much	19.5	108
* Stressed due to eating behavior of the child*		
A little to not at all	96.9	537
Fairly to very much	3.1	17
* Stressed due to sleeping behavior of the child*		
A little to not at all	84.8	470
Fairly to very much	15.2	84

^a^*N* = 972, ^b^*N* = 554 (including only parents of infants)

#### Parental depression and anxiety symptoms

On the State scale of the STADI, 24.1% of the parents showed values ‘above average’ or ‘far above average’ (see Table 5). Looking at the subscales separately, symptoms above or far above average were shown in 18.5% and 30.1% of the sample for depression and anxiety, respectively.

**Table 5:** Parental and child mental health according to the State Scale of STADI (parents), CSF (infants) and SDQ (toddlers)

Parental mental health (STADI)	%	n
* Depression*^*a*^		
Far below average	4.3	42
Below average	25.7	251
Average	51.5	504
Above average	16.6	162
Far above average	1.9	19
* Anxiety*^*b*^		
Far below average	0	0
Below average	9.0	88
Average	60.9	596
Above average	25.9	254
Far above average	4.2	41
* Total*^*a*^		
Far below average	4.2	41
Below average	10.9	107
Average	60.8	595
Above average	20.1	197
Far above average	4.0	39

Child mental health (CSF and SDQ)	%	n
* Regulatory problems (noticeable crying, feeding & sleeping problems)*^*c*^		
Excessive crying (Wessel criterion)	3.2	18
Crying/Whining/Sleeping	26.2	145
Feeding	34.8	193
Multiple regulation problems (crying, sleeping and feeding)	13.5	75
* Emotional and behavioral problems (Categorial evaluation of SDQ total score)*^*d*^		
Inconspicuous/normal	81.5	356
Borderline	10.1	44
Noticeable/abnormal	8.5	37
* Noticeable/abnormal problem scores according to SDQ-subscales*^*d*^		
Emotional problems	6.6	29
Conduct problems	6.2	27
Hyperactivity	6.9	30
Peer problems	7.6	33

^a^*N* = 978, ^b^*N* = 979, ^c^*N* = 554 (infants), ^d^*N* = 437 (toddlers)

#### Infants’ crying, sleeping and feeding problems and toddlers’ emotional and behavioral problems

According to the CSF, 34.8% of the infants showed feeding problems, 26.2% had noticeable crying/sleeping problems, and 13.5% were reported to have multiple regulatory problems (crying, sleeping and feeding) (see Table 5). The SDQ showed 10.1% of the toddlers to be in the ‘borderline’ range and 8.5% in the ‘abnormal’ range with regard to emotional and behavioral problems. In terms of the SDQ-subscales, 7.6% of the parents (*n* = 33) reported peer problems, 6.9% (*n* = 30) hyperactivity, 6.6% (*n* = 29) emotional problems and 6.2% (*n* = 27) conduct problems in their children (see Table 5).

### Correlations between investigated psychosocial stress factors

#### Pandemic burden and EBI, STADI, CSF, SDQ

Moderate significant correlations were identified between the pandemic burden of the parents and EBI total score (ρ = 0.265) respectively STADI total score (ρ = 0.360; mothers only) (*p* < 0.001). Significant weak correlations were detected between parental pandemic burden and CSF crying/sleeping problem score (ρ = 0.136) and CSF feeding problem score (ρ = 0.089) (*p* < 0.05), respectively SDQ total score (ρ = 0.173) (*p* < 0.001). A weak significant correlation between child`s pandemic burden (parent report) and CSF crying/sleeping problem score (ρ = 0.102) respectively SDQ total score (ρ = 0.122) (*p* < 0.05) was found. Among the individual stresses related to the pandemic, ‘increased conflicts in the family’ correlated most strongly with the other outcomes, in detail moderately with EBI total score (ρ = 0.491) and weakly with SDQ total score (ρ = 0.292), STADI total score (ρ = 0.253), CSF crying/sleeping problem score (ρ = 0.229) and CSF feeding problem score (ρ = 0.181) (*p* < 0.05).

#### EBI, STADI and CSF, SDQ

Moderate significant correlations were identified between ‘CSF feeding problem score’ and ‘EBI total score’ (ρ = 0.356) respectively ‘STADI total score’ (ρ = 0.307; mothers only) (*p* < 0.001). Moderate significant correlations were also found between ‘CSF crying/sleeping problem score’ and ‘EBI total score’ (ρ = 0.474) respectively ‘STADI total score’ (ρ = 0.494; mothers only) (*p* < 0.001). ‘SDQ total score’ correlated moderately with ‘EBI total score’ (ρ = 0.419) respectively ‘STADI total score’ (ρ = 0.366; mothers only) (*p* < 0.001).

#### Sociodemographic characteristics predictive of psychosocial stress factors

The logistic regression model (*n* = 906) yielded a perceived higher financial status to make high parental pandemic burden less likely, *OR* = 0.634, 95% CI [0.525; 0.767]. The other predictor variables did not show a significant influence on the parental pandemic burden score in the logistic regression model.

For the EBI total score, the multiple linear regression model (*R*^2^ = 0.04, *F*(4, 904) = 9.44, *p* < 0.001) showed ‘education status’ to have the highest impact (β = 0.137*, p* < 0.001), followed by ‘financial status’ (β = − 0.129*, p* < 0.001) and ‘child age’ (β = 0.083, *p* = 0.005). Having more than one child was not significantly associated with the EBI total score. Accordingly, parents with higher education level and with older children are expected to have a higher EBI total score, whereas a higher financial status predicts a lower EBI total score.

The linear regression model indicated that the STADI total score (*R*^2^ = 0.08, *F*(4, 842) = 18.34, *p* < 0.001) is higher in women with a higher education status (β = 0.083, *p* = 0.014), whereas the score is lower in women with high financial status (β = − 0.187, *p* < 0.001). Increasing child’s age is also significantly associated with a higher STADI total score in women (β = 0.194, *p* < 0.001). Having more than one child was not significantly associated with the STADI total score. The multiple linear regression model for men did not show any significant effect (data not shown).

Regarding the CSF feeding problem score, only ‘education status’ and ‘child gender’ were included as predictor variables in the linear regression model since the other determinants did not comply requirements to be included. The model (*R*^2^ = 0.022, *F*(2, 514) = 5.84, *p* = 0.004) showed a higher parental education status (β = 0.113, *p* = 0.010) and female gender of the child (β = 0.096, *p* = 0.029) to be associated with a higher CSF feeding problem score. For the CSF crying/sleeping problem score, the model (*R*^2^ = 0.016, *F*(2, 514) = 4.22, *p* = 0.015) revealed that having a sibling (β = -0.093, *p* = 0.034) and child’s age (β = 0.092, *p* = 0.036) are associated with CSF crying/sleeping problem score. Accordingly, having a sibling is associated with lower and increased age is associated with higher CSF crying/sleeping problem score.

The predictor variable ‘financial status’ had the highest impact on the SDQ total score (β = − 0.190*, p* ≤ 0.001), followed by ‘education status’ (β = − 0.142*, p* = 0.004), and ‘child age’ (β = 0.104*, p* = 0.031) in the multiple linear regression model (*R*^2^ = 0.07, *F*(3, 405) = 10.72, *p* < 0.001). Thus, children whose parents’ financial status and education status is high are expected to have a lower SDQ total score. Regarding children’s age, the results show that an increasing age in children is associated with a higher SDQ total score.

## Discussion

This cross-sectional survey with almost one thousand participants investigated parent and child psychosocial stress factors in families with children aged 0–3 years during the COVID-19 pandemic in Bavaria, Southern Germany. Our results showed that a large majority of the parents perceive a high pandemic burden. Noticeable values of parenting stress were revealed in well over one third and parental affective symptoms in up to one third of the sample. The evaluation of infant regulatory problems showed a relatively high rate of crying, sleeping and feeding problems compared to pre-pandemic studies, while the percentage of toddlers’ emotional and behavioral problems corresponded to the norm. Children`s mental health problems correlated higher with parenting stress and parental affective symptoms than with perceived pandemic burden. A lower financial status, higher parental education and increasing child age predicted higher parenting stress and affective symptoms as well as higher psychological problems in infants or toddlers.

In detail, the CoronabaBY study revealed 65% of the parents to experience the pandemic as stressful or very stressful, thus perceiving a high pandemic burden. This rate appears to be slightly higher than the ones of other German COVID related studies, where pandemic burden rates between about 50% [48] and 59% [4] in families with children younger than 14 years have been found. While not fully comparable due to individual differences in wording and in the design of the response categories, the assessment of how stressful the pandemic is perceived was also administered with one global question in these comparative studies. Our results indicate that families with infants and toddlers are similarly or even more affected by pandemic-related restrictions as families with older children. Restrictions of leisure activities and limited access to family support services were rated as most stressful. Family support services, e.g., early childhood intervention services, explicitly target families with limited parenting resources as could be found in our sample. This raises the question if psychosocial support measures sufficiently reach families in need during the pandemic.

The overall EBI revealed parenting stress in about 38% of the respondents, indicating a limitation of parental resources in the upbringing and care of the child. Roughly two thirds of all parents showed limited emotional availability in the parent-child relationship (EBI subscale ‘depression’). The rates of parents with scores above cut-off was consistently higher in the EBI subscales ‘role restriction’, ‘isolation’, ‘attachment’, and ‘parental competence’ during the COVID-19 pandemic compared to the pre-pandemic study KiD 0–3 [49]. Also, the percentage of parents (up to 19.5%) who experienced their child’s behavior and symptoms (crying/whining, sleeping) as stressful appeared to be higher than in the previous German cohort study (up to 12.8%) [49].

A closer look at parental mental health revealed that affective symptoms occurred in a quarter of the participants in the overall sample, with symptoms of anxiety being specifically pronounced (over 30%). Comparing the data with the German study KiD 0–3, in which a fifth of the parents with children up to 3 years were detected with depressive/anxious symptoms [50], affective symptoms seem to be more frequent in parents in our sample during the pandemic.

These findings may be regarded as worrying for the following reasons: First, parenting stress and parental affective symptoms are known to have a significant impact on children’s mental health outcomes: Both parameters are directly linked to child emotional and behavior problems [24, 51, 52], which has also been confirmed during the pandemic [16, 17, 25, 28, 32]. Also, parenting stress and parental affective symptoms as found in our study are known to decrease parent-child-interaction quality, e.g., due to limited emotional availability [19–21]. Since a strong positive parent-child relationship is the main contributing factor for children’s resilience (e.g., [53, 54]), this might also add to a possible decrease of child mental health. Second, an accumulation of psychosocial risk factors as present in our sample increases the risk of parental burnout [55] which can at worst result in child neglect or maltreatment [13]. Accordingly, the Federal Statistical Office in Germany reported the highest level of child welfare endangerments in the COVID year 2020 since the introduction of the statistics in 2012 [56].

We found the rate of feeding (34.8%) and crying/sleeping (26.2%) problems as well as multiple regulatory problems (13.5%) in infants (as measured by CSF) to be relatively high in comparison to pre-pandemic reference studies in non-clinical samples, where incidences for all these problems usually range up to around 20% [49, 57–62] with occasional higher rates for specific age groups and corresponding developmental stages during infancy (e.g., [61]). However, comparability between studies is generally limited, since both definition and assessment of infant crying, sleeping and feeding problems vary greatly. Since these problems are known to be very common and often transient in infancy [60, 63], the found rates appear to be within a normal range for our infant sample mean age of 6 months. In addition, since the feeding problem subscale of the CSF has limited predictive values, it is quite possible that the prevalence in this regard is slightly overestimated in our sample. For toddlers, the found rate of 8.5% emotional and behavioral problems (as measured by SDQ total score) corresponded to the norm [46, 64]. The subscale scores were even lower (6.2–7.6%) in comparison to the German COPSY-study, which recorded higher SDQ scores for older children during the pandemic (10–14.6%) [65]. We therefore argue that the rate of mental health problems in very young children in our sample appears overall not noticeably higher than documented in studies before the pandemic.

These results are somewhat surprising with regard to studies that show a significant rise in mental health problems for older children during the pandemic (e.g., [65]). One explanation could be that many infants and toddlers might not yet be directly affected by the COVID restrictions (e.g., by closures of childcare facilities, as the majority of parents in our sample are still on maternal/parental leave). However, what could be expected is a shifted influence of parental psychosocial stress on child symptomatology in the future [35, 49] as young children have the highest dependency on parental care [12].

Within the cohort, parental pandemic burden showed significant weak to moderate correlations with parenting stress (EBI), parental affective symptoms (STADI), infants’ regulatory problems (CSF subscales) and toddlers’ emotional and behavioral problems (SDQ), which could gain further relevance if pandemic conditions persist. Also, since parenting stress and parental mental health showed significant moderate correlations with infants’ regulatory respectively toddlers’ emotional and behavioral problems, the high rates of these parental outcomes are alarming and might foster long-term childhood mental health problems. Overall, child symptoms appear to be more closely related to parenting stress and parental affective symptoms than to actual pandemic-related burden — which might support the hypothesis of an indirect impact on young children via the path of parental psychosocial stresses. However, this assumption is limited by the fact that it was drawn from a correlation design, and no statement can be made about causality. We will address this aspect in a follow-up evaluation of our sample.

With regard to sociodemographic characteristics, we identified the financial and education status and the age of the child as main factors influencing psychosocial stressors. A higher financial status was a potential protective factor regarding parenting stress and parental mental health as well as mental health of toddlers. The strengthening character of a comfortable financial situation with regard to mental health is known from literature [66, 67]. In contrast, higher education was identified as a potential risk factor for parenting stress, parental mental health problems as well as feeding problems in infants. This result might be somewhat surprising with regard to contrary findings of pre-pandemic studies [35, 68] but is well in line with other observations during COVID (e.g., [69]). Reasons could be that highly educated parents are possibly better informed about COVID and therefore more anxious about an infection. Moreover, they probably work more often in jobs which could be shifted to the home office, resulting in having to handle work and childcare simultaneously in case of closed childcare facilities [28]. Increasing child age was linked to more parental affective symptoms and higher parenting stress. Similar relationships were also observed in other studies, as many psychosocial stress factors that affect parents with infants and toddlers increase in the course of early childhood [35]. This might be a reason why increasing child age was also associated with higher scores of infants` crying/sleeping problems and toddlers` emotional and behavioral problems in our sample. However, all of the corresponding effect sizes were small. Therefore, interpretation of these characteristics as risk factors is limited.

 Our results should be seen against the background of the study’s strengths and limitations. To our knowledge, this is the first German study to investigate psychosocial stress factors in families with children aged 0–3 years during the pandemic. The completely digital study process allowed us to examine a large population despite of physical distancing measures. Besides a notably high number of participants, we assessed parenting stress and parental and child mental health with validated standardized psychological questionnaires without notable missing values.

However, there are some limitations with regard to the representativeness of our results. Online samples are often self-selecting (e.g., higher willingness to participate by persons with a higher level of education) [70, 71] and study participation was only possible for app users. Accordingly, our sample had an overall high financial status. Also, the level of education appears to be higher than in the general population of Bavaria [72]. This must be considered relevant because financial and education status were identified as influencing factors for the study outcomes and the sample’s characteristics could have biased the results in a certain direction. However, the level of education in our study population seems to be more evenly distributed than in comparative COVID related online-studies. Since the respondents were almost exclusively mothers, we cannot draw a specific conclusion for fathers. A mere 7.4% to 8.5% of the examined respondents reported characteristics of a migration background (assessed by questions regarding mother tongue and country of birth) — compared to the population of Bavaria, where one third of families has a migration background [73] — likely due to app and questionnaires being only applicable in German. Studies show that this population group carries a higher risk of psychosocial stress and children with migration background were affected significantly more by the pandemic in Germany (65). However, even in our overall well-off sample, psychosocial stress factors were pronounced and can therefore also be expected in potentially less privileged population groups. Due to feasibility reasons, we did not collect non-participants characteristics. We cannot rule out that families with strongly pronounced psychosocial stress factors participated less frequently, thus might be underrepresented in our sample. Of course, all these aspects have to be taken into account with regard to the generalizability of our study results. Nevertheless, the recruitment procedure has enabled us to reach families with young children during times of severe pandemic-related restrictions and our results provide a first insight into their specific psychosocial situation during COVID-19.

Taken together, our findings indicate that parental psychosocial stress factors are strongly pronounced in Bavarian families with children aged 0–3 years during COVID 19 — regarding perceived pandemic burden even more distinct than in families with older children. Parents with very young children are to a great extent emotionally affected, which is especially reflected in limited resources for childcare and in the presence of parental affective symptoms. Overall, the effects of the pandemic seem to not have manifested in very young children’s mental health problems yet. Due to their high dependence on an intact parent-child interaction as well as emotional availability of the parents, staggered detrimental effects on infants’ and toddlers’ healthy development might be expected if the stressful living conditions persist. This assumption is further supported by the significantly moderate correlations already demonstrated between the parental and child psychosocial stress factors. Therefore, secondary prevention efforts like early childhood intervention services that target strengthening parenting skills and the parent-child relationship are more vital than ever.

## Data Availability

All data generated or analyzed during this study are included in this published article.

## Abbreviations

CSF: Questionnaire for Crying, Sleeping and Feeding
EBI: Eltern-Belastungs-Inventar (German version of ‘Parenting Stress Index (PSI)’)
SDQ: Strengths and Difficulties Questionnaire
STADI: State-Trait Anxiety-Depression Inventory
